# Text Mining of United States Obesity-Related Public Policies: Systematic Document Search

**DOI:** 10.2196/13235

**Published:** 2020-07-29

**Authors:** Caroline Spanhol-Finocchio, Mariana de Freitas Dewes, Giana de Vargas Mores, Homero Dewes

**Affiliations:** 1 Federal University of Mato Grosso do Sul Campo Grande, Mato Grosso do Sul Brazil; 2 Federal University of Health Sciences of Porto Alegre Porto Alegre, Rio Grande do Sul Brazil; 3 IMED Business School Passo Fundo, Rio Grande do Sul Brazil; 4 Federal University of Rio Grande do Sul Porto Alegre, Rio Grande do Sul Brazil

**Keywords:** government, data mining, school, health policy

## Abstract

**Background:**

Obesity has become a worldwide health problem, caused by multiple and complex factors. To face this challenge, governments have played a central role in combating its rise. Considering this, public policies are introduced or enacted for the benefit of whole populations, taking into account the perspective of multiverse social stakeholders based on solid scientific fundamentals.

**Objective:**

The aim of this study was to examine obesity-related public policies in the United States and the District of Columbia, in order to understand their scientific basis.

**Methods:**

We analyzed the public policies implemented in the United States from 2003 to 2013, during which time the largest number of obesity-related public policies were introduced, using text mining.

**Results:**

In total, 1592 obesity-related public policies were retrieved from the Centers for Disease Control and Prevention. Multidisciplinary policies were predominant in the documents analyzed (533/1592, 33.5%), followed by health sciences (454/1592, 28.5%), social sciences (330/1592, 20.7%), life sciences (240/1592, 15.1%), and physical sciences (35/1592, 2.2%). Throughout the country, most policies were community oriented (1082/1865, 58.0%) and many of them were related to school and family environments (447/1865, 24.0%), early care and education (75/1865, 4.0%), hospitals (63/1865, 3.4%), and workplaces (47/1865, 2.5%).

**Conclusions:**

The contents of obesity-related public policies were generally uniformly framed across the United States. They were generally based on scientific references, in which there was a predominance of multidisciplinary research. These findings are consistent with what is known about the multiple factors causing obesity and about the methods being developed to control the epidemic.

## Introduction

Obesity is the result of a complex set of interactions among multiple factors, and it is considered a worldwide problem. Due to its established health risks and substantial increases in its prevalence, obesity has become a global health challenge. Based on data from the Organization for Economic Co-operation and Development, across the globe, 19.5% of the adult population was obese in 2015. This rate ranged from less than 6.0% in Korea and Japan to more than 30.0% in Hungary, New Zealand, Mexico, and the United States [1,2].

Projections made by the Organization for Economic Co-operation and Development [1] show that, in the US, Mexico, and England, 47.0%, 39.0%, and 35.0% of the population, respectively, will be obese by 2030. Considering the high obesity rates around the world, many stakeholders, including the public, scientific communities, media, and governments have been involved in finding ways to prevent and control obesity. The growing number of scientific publications on this topic shows its importance. There is an increasing consensus regarding the importance of and urgency in searching for solutions to obesity, which has placed the issue on many countries’ political agendas, as is the case in the US [3].

Nutrition is presented as a challenging issue, requiring an expanded view that demands different theoretical references for its exploration [4]. Obesity also requires broad interdisciplinary analysis and a sustained response from society [5]. Considering these aspects, the formulation of policies is considered more complex due to the multidimensionality of obesity. In order to overcome this difficulty, the scientific basis used in the development of those policies can be studied.

Researchers generally share the view that science should support the elaboration of policies [6-9]. Science should be used to respond to the demands of society and industry as well as to support the government and its political decisions [10,11]. In order to succeed in nutrition-related policies, it is important to have an adequate level of scientific evidence with the objective of avoiding unintended consequences. Scientific inquiry has been used to contribute to the process of nutrition policy making [12].

The US government plays an important role in health promotion and disease prevention among the US population. The states have legislative and regulatory interests that encourage individuals to eat healthy foods and lead active lives; therefore, the state and local governments implement comprehensive and multisectoral solutions to improve the health of their citizens and prevent obesity [13].

The aim of this study was to examine the official obesity-related public policies in all the states and in the District of Columbia using text mining, in order to identify which areas of knowledge have guided the development of these policies. Text mining is a knowledge discovery process that uses data extraction and analysis techniques from texts, phrases, or words. It involves the application of computational algorithms that process text and identify useful and implicit information that could not normally be retrieved using traditional query methods, since they are usually in an unstructured form [14].

In the last few years, technology has improved information readability and accessibility for researchers, patients, governments, health care professionals, and other information consumers.

These technologies can support not only healthcare professionals and patients’ situational awareness and decision making but also knowledge discovery in health science (p 128)

15

Considering these arguments, this study aims to have two main contributions; it will demonstrate a useful analytical framework for identifying patterns and information from a large volume of documents and that the results obtained can guide government investments in science for its potential contribution to the development of policies related to obesity.

## Methods

### Text Mining

Text mining, which involved information retrieval, textual analysis, information extraction, clustering, categorization, visualization, database technology, and data mining, was used [16]. Many studies [17-23] have also used text mining in researching different subjects, including health topics. For example, one study [23] analyzed the characteristics of general public opinion in relation to diabetes, diet, exercise, and obesity expressed on Twitter using a multicomponent semantic and linguistic framework.

**Figure 1:**
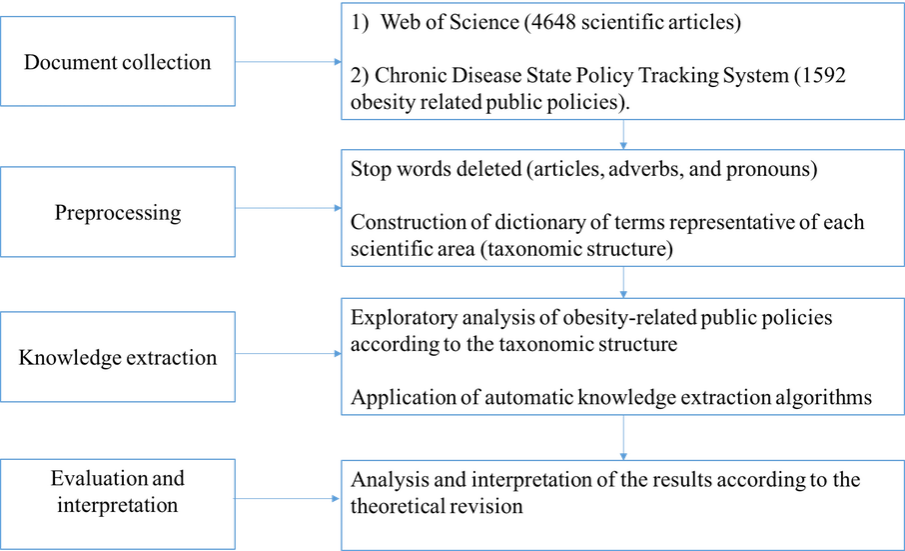
Methodological design.

Text mining can contain several stages; however, some steps are basic in all processes—document collection, preprocessing, knowledge extraction, and evaluation and interpretation of results [24] (Figure 1).

### Document Collection

In the document collection stage, two databases were used. The first was the Web of Science database to search scientific documents. The second was the Chronic Disease State Policy Tracking System to search the summaries of all the obesity-related public policies introduced or established in all states during the period from 2003 to 2013. In that period, we observed greater governmental concern with obesity, and an increased number of public policies.

For scientific articles, we used the set of keywords *food* AND *consumption* AND *obesity* OR obesogen* in Web of Science to search the scientific documents for the period of 2003 to 2013. The words *food* AND *consumption* were chosen in order to capture articles that deal with diets, food consumption, and eating habits. The words *obesity* OR obesogen* were chosen because they were directly related to the research object. In addition, this set of words was submitted for validation by experts on this subject. Content validity was a subjective assessment, usually involving consultation with a small sample of experts to judge the appropriateness of the indicators [25].

For obesity-related public policies, we used the search filters available in the Chronic Disease State Policy Tracking System and selected policies related to nutrition, obesity, and physical activity.

### Preprocessing

In the preprocessing stage, the terms that would be used for the extraction of knowledge were defined. For that, the stop words, terms with no relevant meaning for the research such as articles, adverbs and pronouns were eliminated. In addition, morphological variations were identified using lemmatization.

### Knowledge Extraction

In defining the terms used in the extraction of knowledge, we constructed a taxonomic structure starting with the identification of scientific areas that were found in the obesity-related scientific literature. We organized all the scientific articles according to the journal in which they were published. These journals were subsequently classified according to the scientific area to which they belonged, based on the editorial scope of the journal and established categories in the Web of Science Multimedia Appendix 1. Results were classified into five scientific areas—health sciences, life sciences, physical sciences, social sciences, and multidisciplinary. Complex problems that reached the contemporary scientific agenda generally involved more than one discipline. In this regard, *multidisciplinary* was understood as the possibility of tackling a given subject from multiple viewpoints, encompassing its inherent complexity and extrapolating restrictions related to disciplines [26]. The multidisciplinary approach presented perceptions of two or more disciplines to investigate and solve complex problems [27]. The decision to use these major areas of knowledge was based on the need to improve the explanatory power of the model, namely the taxonomic structure. Thus, we decided to group specific areas (such as pediatrics, general medicine, nursing, etc) due to their similarity, since they belong to the same wide field of health sciences. From that perspective, we considered that a major area of knowledge is made up of disciplines which are similar to each other, but different from other areas.

The next step involved the construction of the dictionary of terms, representative of each scientific area. The list of terms, called *d-words*, was composed of the most relevant keywords that best described the scientific area Multimedia Appendix 2. Each scientific area required a specific list of d-words. Titles, abstracts, and keywords of the scientific articles were inserted in the QDA Miner software (version 3.2; Provalis Research). In order to determine the list of representative words for each scientific area, we identified words that had a higher term frequency–inverse document frequency product. The *term frequency–inverse document frequency product* was used to evaluate how important a word was to a document in a collection. A high term frequency–inverse document frequency product strongly implied relevance of the word to the document and of the document to the scientific area [28]. The term frequency–inverse document frequency product is composed of the normalized *term frequency,* the number of times a word appears in a document divided by the total number of words in that document, and the *inverse document frequency*, the logarithm of the number of documents in the corpus divided by the number of documents where the specific term appears [29].

We ordered the d-words of each scientific area in decreasing value of term frequency–inverse document frequency product and used the first percentile ranking to select the number of specialized words that best characterized the disciplinary dimension. The resulting number of words depended on the criteria established (which were based on our research objectives) [30].

We identified similar words belonging to different subject dimensions. In order to differentiate the similar terms, we used Jaccard coefficients to find the next two terms, which contextualized them in the respective scientific area. This coefficient ranges from 0 to 1 (ie, the closer to 1, the greater the similarity).

The next step was to insert all the obesity-related public policies into the software for knowledge extraction to be performed following the taxonomic structure described. At this step, an exploratory analysis of the data was performed, based on the frequency of words and word expressions. Automatic knowledge extraction algorithms were applied to search for unknown information [22]. The algorithms were used to group similar objects through a measure of proximity. The last step consisted of evaluation and interpretation.

### Evaluation and Interpretation

An exploratory analysis when combined with clustering, allows identifying functional relationships between specific keywords and categories defined by the values of the independent variable. This allows for the visualization of groups of cells with high and low relative frequencies [31].

Clustering analyses were performed directly on the cross-tabulation tables. As a consequence, the similarity index, computed for two keywords or categories and used for clustering, measured the similarity of their distribution among the various groups of the independent variable. A dendrogram was used to visualize how keywords were distributed across the various subgroups such that similar distributions would tend to be grouped under the same cluster [31]. WordStat (Provalis Research) used an average-linkage hierarchical clustering method to create clusters from a similarity matrix. Words or categories that tended to appear together were combined at an early stage, while those that were independent of one another tended to be combined at the end of the agglomeration process [31].

## Results

We obtained 131 d-words for health sciences, 92 d-words for life sciences, 72 d-words for multidisciplinary sciences, 55 d-words for social sciences, and 28 d-words for physical sciences. In total, 4648 scientific articles and 1592 obesity-related public policies were analyzed.

The Call to Action to Prevent and Decrease Overweight and Obesity, promoted by the US Surgeon General in 2001, identified obesity as a key public health priority for the US [32]. Most of the policies were concentrated in the years 2009, 2010, and 2011, representing 62.8% (1000/1592) of the total obesity-related public policies analyzed. For example, one of the topics was improving food environments in schools and childcare settings. After 2009, we noted the inclusion of different topics dealing with obesity. These were associated with governmental priorities in this period [33-35].

During the period from 2003 to 2013, the analysis by state showed that Texas had the highest number of obesity-related public policies (101/1592, 6.3%), followed by California (82/1592, 5.2%), Illinois (79/1592, 5.0%), Maryland (70/1592, 4.4%), Arkansas (52/1592, 3.3%), and New York (51/1592, 3.2%), while South Carolina (9/1592, 0.6%), Kansas (8/1592, 0.5%), Alaska (8/1592, 0.5%), Wyoming (7/1592, 0.4%), and South Dakota (5/1592, 0.3%) were among the states that had the lowest number of policies related to obesity.

According to Behavioral Risk Factor Surveillance System data published in 2018 [34], adult obesity rates exceeded 35.0% in 7 US States—Iowa, Oklahoma, Arkansas, Louisiana, Mississippi, Alabama, and West Virginia. Considering the number of obesity-related public policies, these states did not have a large number of policies from 2003 to 2013; Iowa (23/1592, 1.4%), Oklahoma (37/1592, 2.3%), Mississippi (43/1592, 2.7%), Alabama (14/1592, 0.9%), and West Virginia (20/1592, 1.3%) had relatively low numbers of policies. Only Arkansas (52/1592, 3.3%), Louisiana (51/1592, 3.2%), and New York (51/1592, 3.2%) had more than 50 obesity-related public policies. Even the states with the highest number of obesity-related public policies had high rates of obesity. For example, in Texas (highest number of policies), Illinois (third highest number of policies), Maryland (fourth highest number of policies), and Arkansas (fifth highest number of policies), adult obesity rates exceeded 30.0% [36].

In order to understand the complexity and multidimensionality of this issue, it was necessary to know the content and amount of obesity-related public policies. Table 1 shows that most obesity-related public policies were community oriented, followed by those related to school and after school environments, restaurants and food retail, early care and education, medical facilities and hospitals, and lastly, workplace environments.

**Table 1:** Number of obesity-related public policies in US states (2003-2013).

Setting^a^	Number of policies
Community	1082
School and after school	447
Restaurant and food retail	151
Early care and education	75
Medical facilities and hospital	63
Workplace	47
Total^b^	1865

^a^Elaborated by the authors based on Centers for Disease Control and Prevention [37].

^b^The sum is greater than the number of obesity-related public policies analyzed (1592) because the Centers for Disease Control and Prevention has framed some policies in more than one category.

Most policies were concentrated within the community setting, which included different environments in which people live such as neighborhoods, schools, workplaces, play areas, and places of worship. The content of policies directed to restaurants or food retail locations included menu labeling, access to healthy foods, and food produced locally.

Most of the policies that were analyzed were directed toward modifying environmental factors with the aim of making the environment less obesogenic. We noted that many studies and reports from influential health organizations call on policy and population-based approaches to change the obesogenic environment to combat the obesity epidemic [38]. A number of authors have investigated the influence of the environment on obesity, with some mentioning the influence of fast food in food habits and weight gain [39-41].

**Figure 2:**
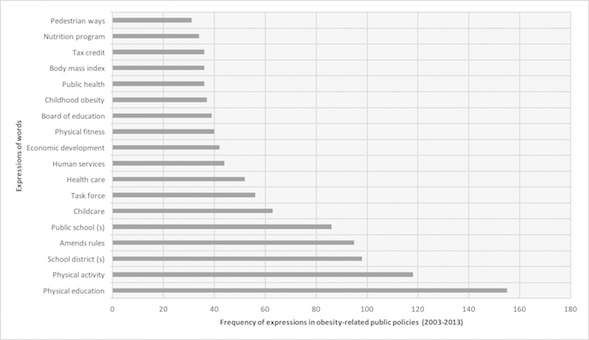
The most frequent obesity-related public policy expressions in US states and the District of Columbia.

With the objective of identifying the most frequent expressions of words in policy content, we selected frequent expressions with a minimum of two and a maximum of four words. This showed which words were more frequent in the set of documents and their focus over time. The most frequent expressions in obesity related public policies included: “physical education” (155/1592, 9.73%), “physical activity” (118/1592, 7.41%), “school district(s)” (98/1592, 6.15%), “amends rules” (95/1592, 5.96%), and “public school(s)” (86/1592, 5.4%). Expressions oriented to early childhood care were also highlighted, as evidenced by the presence of expressions such as “childcare” (63/1592, 3.95%) and “childhood obesity” (37/1592, 2.32%). Moreover, we found expressions related to the practice of physical activities such as “physical fitness” (40/1592, 2.51%) and “pedestrian ways” (31/1592, 1.94%) (Figure 2).

We noted that the expression “amends rules” started to appear (2/1592, 0.12%) in 2008. Similar behavior was observed in the expressions “nutrition program” (11/1592, 0.69%) and “pedestrian ways” (1/1592, 0.06%) in 2007. “Childcare” appeared with more frequency (17/1592, 1.06%) in 2010. On the other hand, the expressions “physical activity” (mean 10.72, range 1-18) and “physical education” (mean 14.09, range 5-27%) have been used since 2003.

After identifying the most relevant expressions in obesity-related public policies, we attempted to identify how science was expressed in the content of these policies. Using the taxonomic structure, the multidisciplinary sciences represented 33.5% (533/1592) of the content of the documents analyzed, followed by health sciences (454/1592, 28.5%), social sciences (330/1592, 20.7%), life sciences (240/1592, 15.1%), and physical sciences (35/1592, 2.2%).

A detailed analysis of the clusters using the Jaccard coefficient (Table 2) showed the existence of a greater similarity between health sciences and life sciences (Jaccard coefficient 0.672) and between multidisciplinary sciences and health sciences (Jaccard coefficient 0.649). It may be explained by the fact that knowledge produced by life sciences is applied in health sciences. The multidisciplinary sciences area is closer to health sciences and life sciences because it gathers knowledge from both areas. Social sciences have a moderated similarity with health sciences and life sciences, whereas physical sciences have a lower similarity between all areas.

**Table 2:** Jaccard coefficient between the scientific areas expressed in obesity-related public policies in the US.

Scientific areas	Health sciences	Life sciences	Multidisciplinary sciences	Physical sciences	Social sciences
Health sciences	1	0.672	0.649	0.134	0.591
Life sciences	0.672	1	0.561	0.180	0.572
Multidisciplinary sciences	0.649	0.561	1	0.116	0.508
Physical sciences	0.134	0.180	0.116	1	0.124
Social sciences	0.591	0.572	0.508	0.124	1

**Figure 3:**
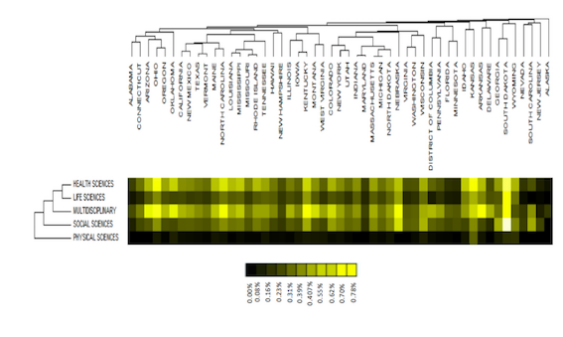
Relationships between obesity-related public policies in US states and scientific areas.

The relative frequency results of the taxonomic classification by state are shown using heatmaps with a clustering of rows and columns. The brightest colors represent the highest frequencies (Figure 3). This analysis in Figure 3 also shows that all the states responded to obesity in a similar way, with few differences in the scientific frameworks adopted by local specificities. In some states, multidisciplinary sciences predominated with Nebraska, Montana, Ohio, and Oregon as examples. The health sciences category appeared more frequently in Kansas, Oklahoma, Idaho, and North Carolina. The social sciences category predominated in South Dakota, Wisconsin, and Nebraska. Kansas and Indiana were among the states with the greatest number of policies focused on life sciences. On the other hand, the physical sciences category was the scientific area with the lowest relative frequency in the US.

The results in Figure 3 also showed high similarity in the obesity-related public policy content of most states analyzed, such Texas and Vermont, Louisiana and Mississippi, New York and Utah, Indiana and Maryland, Massachusetts and Michigan, and Missouri and Rhode Island. In other words, the content of policies in these states was made in a similar way, considering the scientific fundamentals.

## Discussion

The results showed that obesity-related public policy contents were generally uniformly framed across the US. They were approximately based on the same scientific references in which there was a predominance of the multidisciplinary area. These findings were consistent with what has been discussed with respect to the multifactorial causes of obesity as well as the means to control the epidemic. The high frequency of multidisciplinary sciences in the content of obesity-related public policies (533/1592, 33.5%) supports the findings of various previous studies [4,9,42,43]. In these studies, the authors highlighted that obesity requires a multidisciplinary analysis to be understood and our study shows that the government has used multidisciplinary sciences to address obesity.

We observed that the US obesity-related public policies varied in number, but had similar scientific content. Policies based on advances in scientific knowledge can influence the improvement of well-being and the reduction of population obesity and health expenditures; however, the implementation of public policies can be affected by a wide range of factors that challenge their effectiveness. For example, government influences, other interest groups, limitations imposed by the legislative body, the media, or the public [6]. Based on this, we suggest that new studies should be developed to better understand the factors that may limit the effectiveness of health-related public policy.

We also suggest scanning policies for other d-words, with the intention of verifying the intensity of use and the evolution of use of certain expressions within the policies. Associated with this, the elaboration of indicators to measure the influence of this content on obesity indices would allow for a more in-depth view of the functioning and impact of the content. The use of this methodology, combined with other research techniques, may offer a relevant understanding of how science and government are interrelated. Studies that aim to analyze the association between the number of public policies and the rates of obesity in different states can be useful in the identification of policy effectiveness.

## Appendix

Multimedia Appendix 1 Distribution of scientific papers according to the major scientific areas (2003-2013).

Multimedia Appendix 2 D-words corresponding to the major scientific areas contextualized by the respective associated terms.
