# Supplementary material for: Text Mining of United States Obesity-Related Public Policies: Systematic Document Search
Source: JMIR Public Health Surveill. 2020 Jul 29;6(3):e13235. doi: 10.2196/13235 (PMC7424466; doi:10.2196/13235)
Supplement: Multimedia Appendix 1 [file publichealth_v6i3e13235_app1.docx]

Distribution of scientific papers according to the major scientific areas (2003-2013)

| **Scientific areas** | **Disciplinary areas** | **Number of Journals** | **Frequency of Articles** | **Frequency (%)** |
| --- | --- | --- | --- | --- |
| Health Sciences | General Medicine | 162 | 904 | 19.45 |
|  | Public Health | 29 | 496 | 10.67 |
|  | Paediatrics | 27 | 184 | 3.96 |
|  | Nutrition | 46 | 1,312 | 28.23 |
|  | Nursing | 5 | 12 | 0.26 |
|  | Dentistry | 1 | 4 | 0,09 |
|  | **Subtotal** | **270** | **2,912** | **62.65** |
| Multidisciplinary Sciences | Multidisciplinary | 45 | 817 | 17.58 |
|  | **Subtotal** | **45** | **817** | **17.58** |
| Life Sciences | Agriculture | 23 | 111 | 2.39 |
|  | Bioscience | 38 | 212 | 4.56 |
|  | Neuroscience | 30 | 210 | 4.52 |
|  | Pharmacy | 14 | 68 | 1.46 |
|  | **Subtotal** | **105** | **601** | **12.93** |
| Social Sciences | Business | 9 | 49 | 1.05 |
|  | Economy | 11 | 52 | 1.12 |
|  | Psychology | 17 | 84 | 1.81 |
|  | Law | 2 | 4 | 0.09 |
|  | Social Sciences | 15 | 67 | 1.44 |
|  | **Subtotal** | **54** | **256** | **5.51** |
| Physical Sciences | Environmental Science | 8 | 62 | 1.33 |
|  | **Subtotal** | **8** | **62** | **1.33** |
|  | **Total** | **482** | **4,648** | **100.00** |

Source: Web of Science
