# Supplementary material for: Text Mining of United States Obesity-Related Public Policies: Systematic Document Search
Source: JMIR Public Health Surveill. 2020 Jul 29;6(3):e13235. doi: 10.2196/13235 (PMC7424466; doi:10.2196/13235)
Supplement: Multimedia Appendix 2 [file publichealth_v6i3e13235_app2.docx]

**Appendix A**

*D-words* corresponding to the major scientific areas contextualized by the respective associated terms

**Health Sciences**

| • @ACTIVITY [ACTIVITY AND PHYSICAL \| ACTIVITY AND WEIGHT /P] |
| --- |
| • @ADIPOSE [ADIPOSE AND TISSUE \| ADIPOSE AND EXPRESSION /P] |
| • @ADVERTISING_HEALTH_SCIENCE [ADVERTISING AND TV \| ADVERTISING AND FOOD /P] |
| • @APPETITE_HEALTH_SCIENCE [APPETITE AND TEST \| APPETITE AND REGULATION /P] |
| • @AVAILABILITY_HEALTH_SCIENCE [AVAILABILITY AND ACCESS \| AVAILABILITY AND FOOD /P] |
| • @BALANCE_HEALTH_SCIENCE [BALANCE AND ENERGY \| BALANCE AND APPETITE /P] |
| • @BEHAVIOR_HEALTH_SCIENCE [BEHAVIOR OR BEHAVIORS \| BEHAVIOR AND FEEDING /P] |
| • @BEHAVIOUR_HEALTH_SCIENCE [BEHAVIOUR OR BEHAVIOURS \| BEHAVIOUR AND DIETARY /P] |
| • @BEVERAGE_HEALTH_SCIENCE [BEVERAGE OR BEVERAGES \| BEVERAGE AND SWEETENED /P] |
| • @BLOOD [BLOOD AND PRESSURE \| BLOOD AND RESPONSE /P] |
| • @BMI_HEALTH_SCIENCE [BMI AND BODY \| BMI AND MASS /P] |
| • @BODY_HEALTH_SCIENCE [BODY AND WEIGHT \| BODY AND MASS /P] |
| • @BRAIN [BRAIN AND HYPOTHALAMUS \| BRAIN AND CENTRAL /P] |
| • @BREAKFAST [BREAKFAST AND EATING \| BREAKFAST AND CONSUMPTION /P] |
| • @CALCIUM [CALCIUM AND VITAMIN \| CALCIUM AND IRON /P] |
| • @CANCER [CANCER OR CANCERS \| CANCER AND OBESOGENESIS /P] |
| • @CARBOHYDRATE [CARBOHYDRATE AND PROTEIN \| CARBOHYDRATE AND FAT /P] |
| • @CARDIOVASCULAR [CARDIOVASCULAR AND DISEASE \| CARDIOVASCULAR AND ADHERENCE /P] |
| • @CHANGE [CHANGE AND BEHAVIORS \| CHANGE AND VEGETABLE /P] |
| • @CHILDHOOD_HEALTH_SCIENCE [CHILDHOOD AND SPATIAL \| CHILDHOOD AND PLACE /P] |
| • @CHOLESTEROL [CHOLESTEROL AND LIPOPROTEIN \| CHOLESTEROL AND FASTING /P] |
| • @CHRONIC [CHRONIC AND ADIPOSE \| CHRONIC AND TISSUE /P] |
| • @COMPOSITION [COMPOSITION AND FAT \| COMPOSITION AND BODY /P] |
| • @CONSUMERS [CONSUMERS AND DECISIONS \| CONSUMERS AND FOOD /P] |
| • @CONSUMPTION_HEALT_SCIENE [CONSUMPTION AND ENERGY \| CONSUMPTION AND FOOD /P] |
| • @CONTROL_HEALTH_SCIENCE [CONTROL OR CONTROLS \| CONTROL AND FED /P] |
| • @DAILY [DAILY AND DAY \| DAILY AND STUDENTS /P] |
| • @DAIRY [DAIRY AND PRODUCTS \| DAIRY AND FRUITS /P] |
| • @DENSITY [DENSITY AND ENERGY \| DENSITY AND FOODS /P] |
| • @DEVELOPMENT [DEVELOPMENT AND FED \| DEVELOPMENT AND OBESOGENIC /P] |
| • @DIABETES_HEALTH_SCIENCE [DIABETES AND INSULIN \| DIABETES AND RESISTANCE/P] |
| • @DIET_HEALTH_SCIENCE [DIET AND FED \| DIET AND FAT /P] |
| • @DIETARY_HEALTH_SCIENCE [DIETARY AND INTAKE \| DIETARY AND DIET /P] |
| • @DISEASE_HEALTH_SCIENCE [DISEASE AND CARDIOVASCULAR \| DISEASE AND RISK /P] |
| • @DRINKS_HEALTH_SCIENCE [DRINKS OR DRINK \| DRINKS AND SOFT /P] |
| • @ECONOMIC [ECONOMIC AND SOCIO \| ECONOMIC AND POVERTY /P] |
| • @EDUCATION [EDUCATION AND WEALTH \| EDUCATION AND INCOME /P] |
| • @ENDOCRINOLOGY [ENDOCRINOLOGY AND OBESOGEN \| ENDOCRINOLOGY AND ORGANOTINS /P] |
| • @ENERGY_HEALTH_SCIENCE [ENERGY AND EXPENDITURE \| ENERGY AND INTAKE /P] |
| • @ENVIRONMENT_HEALTH_SCIENCE [ENVIRONMENT OR ENVIRONMENTS \| ENVIRONMENT AND NEIGHBORHOOD /P] |
| • @ENVIRONMENTAL [ENVIRONMENTAL AND FACTORS \| ENVIRONMENTAL AND CORRELATES /P] |
| • @EXPOSURE [EXPOSURE AND DIET \| EXPOSURE AND FOOD /P] |
| • @EXPRESSION [EXPRESSION AND EXPOSED \| EXPRESSION AND BRAND /P] |
| • @FAMILY [FAMILY AND EDUCATIONAL \| FAMILY AND FACTORS /P] |
| • @FAST_HEALTH_SCIENCE [FAST AND FOOD \| FAST AND RESTAURANTS /P] |
| • @FAT_HEALTH_SCIENCE [FAT AND DIET \| FAT AND FED /P] |
| • @FATTY_HEALTH_SCIENCE [FATTY AND ACIDS \| FATTY AND OXIDATION /P] |
| • @FED [FED AND INSULIN \| FED AND EXPRESSION /P] |
| • @FIBER [FIBER AND INSOLUBLE \| FIBER AND CEREAL /P] |
| • @FOOD_HEALTH_SCIENCE [FOOD OR FOODS \| FOOD AND CONSUMPTION /P] |
| • @FREQUENCY [FREQUENCY AND BREAKFAST \| FREQUENCY AND CATEGORIES /P] |
| • @FRUIT_HEALTH_SCIENCE [FRUIT OR FRUITS \| FRUIT AND VEGETABLES /P] |
| • @GASTRIC [GASTRIC AND BYPASS \| GASTRIC AND EMPTYING /P] |
| • @GENE_HEALTH_SCIENCE [GENE AND EXPRESSION \| GENE AND PROFILE /P] |
| • @HABITS_HEALTH_SCIENCE [HABITS AND FOOD \| HABITS AND DIETARY /P] |
| • @HEALTH_HEALTH_SCIENCE [HEALTH AND PUBLIC \| HEALTH AND OBESITY /P] |
| • @HEALTHY_HEALTH_SCIENCE [HEALTHY AND EATING \| HEALTHY AND VEGETABLES /P] |
| • @HEPATIC_HEALTH_SCIENCE [HEPATIC AND METABOLISM \| HEPATIC AND LIPID /P] |
| • @HOME_HEALTH_SCIENCE [HOME AND MEALS \| HOME AND CONSUMED /P] |
| • @HYPERTENSION [HYPERTENSION AND HEART \| HYPERTENSION AND RISK /P] |
| • @HYPOTHALAMIC [HYPOTHALAMIC AND NEURONS \| HYPOTHALAMIC AND NEUROPEPTIDE /P] |
| • @INCOME_HEALTH_SCIENCE [INCOME AND EDUCATION \| INCOME AND HOUSEHOLD /P] |
| • @INDEX_HEALTH_SCIENCE [INDEX AND BODY & INDEX AND MASS /P] |
| • @INSULIN_HEALTH_SCIENCE [INSULIN AND RESISTANCE \| INSULIN AND FED /P] |
| • @INTAKE_HEALTH_SCIENCE [INTAKE OR INTAKES \| INTAKE AND ENERGY /P] |
| • @INTERVENTION_HEALTH_SCIENCE [INTERVENTION AND CONTROL \| INTERVENTION AND TEACHERS /P] |
| • @JUICE [JUICE AND FRUIT \| JUICE AND BEVERAGE /P] |
| • @KNOWLEDGE [KNOWLEDGE AND PREFERENCES \| KNOWLEDGE AND BELIEFS /P] |
| • @LEAN [LEAN AND PHENOTYPE \| LEAN AND EXPENDITURE /P] |
| • @LIFESTYLE_HEALTH_SCIENCE [LIFESTYLE AND SKIPPING \| LIFESTYLE AND BREAKFAST /P] |
| • @LIVER [LIVER AND HEPATIC \| LIVER AND MUSCLE /P] |
| • @LUNCH [LUNCH AND BREAKFAST \| LUNCH AND DRINK /P] |
| • @MASS [MASS AND INDEX \| MASS AND BODY /P] |
| • @MATERNAL [MATERNAL AND OFFSPRING \| MATERNAL AND PREGNANCY /P] |
| • @MEAL [MEAL OR MEALS \| MEAL AND FREQUENCY / |
| @MEDITERRANEAN [MEDITERRANEAN AND ADHERENCE \| MEDITERRANEAN AN |
| • @METABOLIC [METABOLIC AND INSULIN \| METABOLIC AND FAT /P] |
| • @METABOLISM [METABOLISM AND PHYSIOLOGY \| METABOLISM AND ENDOCRI |
| • @MILK [MILK AND PRODUCT \| MILK AND DAIRY /P] |
| • @MOTHERS [MOTHERS OR MOTHER \| MOTHERS AND PARENTING /P] |
| • @MUSCLE [MUSCLE AND TISSUE \| MUSCLE AND SKELETAL /P] |
| • @NUTRIENT [NUTRIENT AND FOOD \| NUTRIENT AND CONSUMPTION /P] |
| • @NUTRITION_HEALTH_SCIENCE [NUTRITION AND INFORMATION \| NUTRITION A |
| • @NUTRITIONAL_HEALTH_SCIENCE [NUTRITIONAL AND EDUCATION \| NUTRITIO |
| • @OBESE [OBESE AND WEIGHT \| OBESE AND BODY /P] |
| • @OBESITY_HEALTH_SCIENCE [OBESITY AND DIET \| OBESITY AND CONSUMPTI |
| • @OBESOGENIC [OBESOGENIC AND FED \| OBESOGENIC AND EXPRESSION /P] |
| • @OFFSPRING [OFFSPRING AND RISK \| OFFSPRING AND OBESITY /P] |
| • @OVERWEIGHT_HEALTH_SCIENCE [OVERWEIGHT AND RISK \| OVERWEIGHT A |
| • @PARENTS_HEALTH_PARENTS [PARENTS AND CHILD \| PARENTS AND BEHAVIO |
| • @PATTERN_HEALTH_SCIENCE [PATTERN AND PROCESSED \| PATTERN AND SM |
| • @PHYSICAL_HEALTH_SCIENCE [PHYSICAL AND ACTIVITY \| PHYSICAL AND BEH |
| • @PLAQUE [PLAQUE AND ACIDITY \| PLAQUE AND ACIDOGENECITY /P] |
| • @POLICY_HEALTH_SCIENCE [POLICY AND PUBLIC \| POLICY AND OBESITY /P] |
| • @POPULATION_HEALTH_SCIENCE [POPULATION OR POPULATIONS \| POPULAT |
| • @PORTION_HEALTH_SCIENCE [PORTION AND SIZE \| PORTION AND SIZES /P] |
| • @PRACTICES_HEALTH_SCIENCE [PRACTICES AND INCLUDE \| PRACTICES AND |
| • @PREVENTION_HEALTH_SCIENCE [PREVENTION AND OVERWEIGHT \| PREVEN |
| • @PRODUCTS_HEALTH_SCIENCE [PRODUCTS AND FOOD \| PRODUCTS AND BEV |
| • @PROTEIN_HEALTH_SCIENCE [PROTEIN AND EXPRESSION \| PROTEIN AND REC |
| • @QUALITY_HEALTH_SCIENCE [QUALITY AND DIET \| QUALITY AND POOR /P] |
| • @RECEPTOR_HEALTH_SCIENCE [RECEPTOR AND EXPRESSION \| RECEPTOR A |
| • @REGULATION_HEALTH_SCIENCE [REGULATION AND ENERGY \| REGULATION |
| • @RESISTANCE_HEALTH_SCIENCE [RESISTANCE AND INSULIN \| RESISTANCE A |
| • @RESPONSE [RESPONSE AND DIETARY \| RESPONSE AND DIET /P] |
| • @RESTAURANT [RESTAURANT OR RESTAURANTS \| RESTAURANT AND MENU / |
| • @RISK_HEALTH_SCIENCE [RISK AND FACTORS \| RISK AND DISEASE /P] |
| • @ROLE [ROLE AND REGULATION \| ROLE AND ENERGY /P] |
| • @SATIETY [SATIETY AND HUNGER \| SATIETY AND EFFECTS /P] |
| • @SCHOOL_HEALTH_SCIENCE [SCHOOL OR SCHOOLS & SCHOOL AND STUDEN |
| • @SIZE_HEALTH_SCIENCE [SIZE AND PORTION \| SIZE AND SERVED /P] |
| • @SLEEP_HEALTH_SCIENCE [SLEEP AND DURATION \| SLEEP AND DEPRIVATION |
| • @SNACKS_HEALTH_SCIENCE [SNACKS OR SNACK \| SNACKS AND UNHEALTHY |
| • @SOCIAL_HEALTH_SCIENCE [SOCIAL AND INFLUENCE \| SOCIAL AND ENVIRON |
| • @SOCIO_HEALTH_SCIENCE [SOCIO AND DEMOGRAPHIC \| SOCIO AND ECONOM |
| • @SOFT_HEALTH_SCIENCE [SOFT AND DRINK \| SOFT AND DRINKS /P] |
| • @STRESS_HEALTH_SCIENCE [STRESS AND OXIDATIVE \| STRESS AND FAT /P] |
| • @SUGAR_HEALTH_SCIENCE [SUGAR OR SUGARS \| SUGAR AND SWEETENED / |
| • @SUGARY [SUGARY AND ALCOHOLIC \| SUGARY AND DRINKS /P] |
| • @SWEETENED_HEALTH_SCIENCE [SWEETENED AND SUGAR \| SWEETENED AN |
| • @SYNDROME [SYNDROME AND METABOLIC \| SYNDROME AND COMPONENTS |
| • @TELEVISION_HEALTH_SCIENCE [TELEVISION AND INTERRELATIONSHIPS \| TE |
| • @TISSUE [TISSUE OR TISSUES \| TISSUE AND ADIPOSE /P] |
| • @TREATMENT [TREATMENT AND OBESITY \| TREATMENT AND PREVENTION /P] |
| • @TV [TV AND VIEWING \| TV AND HABITS /P] |
| • @VEGETABLE [VEGETABLE OR VEGETABLES \| VEGETABLE AND CONSUMPTION |
| • @VIEWING [VIEWING AND TELEVISION \| VIEWING AND TV /P] |
| • @VITAMIN [VITAMIN AND FOLATE \| VITAMIN AND CALCIUM /P] |
| • @WAIST [WAIST AND CIRCUMFERENCE \| WAIST AND MASS /P] |
| • @WATER [WATER AND EXPERIMENT \| WATER AND STUDIED /P] |

**Life Sciences**

| @ACCESS_LIFE_SCIENCE [ACCESS AND STANDARD \| ACCESS AND CALORIES /P] |
| --- |
| • @ACTIVITY_LIFE_SCIENCE [ACTIVITY AND PHYSICAL \| ACTIVITY AND CONSUMPTION /P] |
| • @ADDICTION_LIFE_SCIENCE [ADDICTION AND DEPENDENCE \| ADDICTION AND CRAVING /P] |
| • @ADIPOSE_LIFE_SCIENCE [ADIPOSE AND TISSUE \| ADIPOSE AND WHITE /P] |
| • @ADIPOSITY_LIFE_SCIENCE [ADIPOSITY AND OBESOGENIC \| ADIPOSITY AND GENE /P] |
| • @ANTAGONIST_LIFE_SCIENCE [ANTAGONIST AND RECEPTOR \| ANTAGONIST AND CANNABINOID /P] |
| • @APPETITE_LIFE_SCIENCE [APPETITE AND CONTROL \| APPETITE AND EFFECTS /P] |
| • @BALANCE_LIFE_SCIENCE [BALANCE AND ENERGY \| BALANCE AND LOCAL /P] |
| • @BEVERAGES_LIFE_SCIENCE [BEVERAGES AND SWEETENED \| BEVERAGES AND SUGAR /P] |
| • @BODY_LIFE_SCIENCE [BODY AND WEIGHT \| BODY AND MASS /P] |
| • @BRAIN_LIFE_SCIENCE [BRAIN AND CONTROL \| BRAIN AND FOOD /P] |
| • @CALCIUM_LIFE_SCIENCE [CALCIUM AND DAIRY \| CALCIUM AND RESTRICTION /P] |
| • @CALORIC_LIFE_SCIENCE [CALORIC AND INTAKE \| CALORIC AND DENSE /P] |
| • @CANNABINOID_LIFE_SCIENCE [CANNABINOID AND RECEPTOR \| CANNABINOID AND INVERSE /P] |
| • @CARBOHYDRATE_LIFE_SCIENCE [CARBOHYDRATE AND PROPORTION \| CARBOHYDRATE AND FAT /P] |
| • @CELL_LIFE_SCIENCE [CELL OR CELLS \| CELL AND RESISTANCE /P] |
| • @CHOLESTEROL_LIFE_SCIENCE [CHOLESTEROL AND TOTAL \| CHOLESTEROL AND TRIGLYCERIDES /P] |
| • @CHRONIC_LIFE_SCIENCE [CHRONIC AND ACUTE \| CHRONIC AND THERAPEUTIC /P] |
| • @COMPOUNDS_LIFE_SCIENCE [COMPOUNDS AND BIOACTIVE \| COMPOUNDS AND ANTIOXIDANT /P] |
| • @CONTROL_LIFE_SCIENCE [CONTROL AND CONSUMPTION \| CONTROL AND WEIGHT /P] |
| • @CUES_LIFE_SCIENCE [CUES OR CUE \| CUES AND PREFRONTAL /P] |
| • @DAIRY_LIFE_SCIENCE [DAIRY AND PRODUCTS \| DAIRY AND CALCIUM /P] |
| • @DEVELOPMENT_LIFE_SCIENCE [DEVELOPMENT AND METABOLIC \| DEVELOPMENT AND INSULIN /P] |
| • @DIABETES_LIFE_SCIENCE [DIABETES AND CARDIOVASCULAR \| DIABETES AND DISEASE/P] |
| • @DIETARY_LIFE_SCIENCE [DIETARY AND FAT \| DIETARY AND INTAKE /P] |
| • @DIET_LIFE_SCIENCE [DIET OR DIETS \| DIET AND FAT /P] |
| • @DOPAMINE_LIFE_SCIENCE [DOPAMINE AND REWARD \| DOPAMINE AND DOPAMINERGIC /P] |
| • @DOSE_LIFE_SCIENCE [DOSE OR DOSES \| DOSE AND REDUCED /P] |
| • @DRINKS_LIFE_SCIENCE [DRINKS AND SOFT \| DRINKS AND CARBONATED /P] |
| • @ENERGY_LIFE_SCIENCE [ENERGY AND INTAKE \| ENERGY AND OVERWEIGHT /P] |
| • @EXPENDITURE_LIFE_SCIENCE [EXPENDITURE AND ENERGY \| EXPENDITURE AND ADIPOSE /P] |
| • @EXPOSURE_LIFE_SCIENCE [EXPOSURE AND DIET \| EXPOSURE AND CHRONIC /P] |
| • @EXPRESSION_LIFE_SCIENCE [EXPRESSION AND ARCUATE \| EXPRESSION AND HYPOTHALAMIC /P] |
| • @FAT_LIFE_SCIENCE [FAT AND DIET \| FAT AND DIETARY /P] |
| • @FATTY_LIFE_SCIENCE [FATTY AND ACIDS \| FATTY AND OXIDATION /P] |
| • @FED_LIFE_SCIENCE [FED AND PURIFIED \| FED AND OBESOGENIC /P] |
| • @FETAL_LIFE_SCIENCE [FETAL AND FETUSES \| FETAL AND MATERNAL /P] |
| • @FOOD_LIFE_SCIENCE [FOOD OR FOODS \| FOOD AND WEIGHT /P] |
| • @GENE_LIFE_SCIENCE [GENE OR GENES \| GENE AND EXPRESSION /P] |
| • @GLYCEMIC_LIFE_SCIENCE [GLYCEMIC AND LOAD \| GLYCEMIC AND INDEX /P] |
| • @GUIDELINES_LIFE_SCIENCE [GUIDELINES AND EXPOSED \| GUIDELINES AND ALARMING /P] |
| • @HEALTH_LIFE_SCIENCE [HEALTH AND CONSUMPTION \| HEALTH AND OBESITY /P] |
| • @HISTAMINE_LIFE_SCIENCE [HISTAMINE AND ANTIOBESITY \| HISTAMINE AND RELEASE /P] |
| • @HYPOTHALAMIC_LIFE_SCIENCE [HYPOTHALAMIC AND HYPOTHALAMUS \| HYPOTHALAMIC AND EXPRESSION /P] |
| • @HYPOTHALAMUS_LIFE_SCIENCE [HYPOTHALAMUS AND NUCLEUS \| HYPOTHALAMUS AND HYPOTHALAMIC /P] |
| • @INSULIN_LIFE_SCIENCE [INSULIN AND RESISTANCE \| INSULIN AND DIET /P] |
| • @INTAKE_LIFE_SCIENCE [INTAKE AND ENERGY \| INTAKE AND CONSUMPTION /P] |
| • @JUICE_LIFE_SCIENCE [JUICE AND RESPONSES \| JUICE AND FORTIFICATION /P] |
| • @MASS_LIFE_SCIENCE [MASS AND INDEX \| MASS AND BODY /P] |
| • @MATERNAL_LIFE_SCIENCE [MATERNAL AND OFFSPRING \| MATERNAL AND FETAL /P] |
| • @MEAL_LIFE_SCIENCE [MEAL AND HUNGER \| MEAL AND CONDITION /P] |
| • @MECHANISMS_LIFE_SCIENCE [MECHANISMS AND METABOLIC \| MECHANISMS AND FAT /P] |
| • @METABOLIC_LIFE_SCIENCE [METABOLIC AND SYNDROME \| METABOLIC AND FAT /P] |
| • @METABOLISM_LIFE_SCIENCE [METABOLISM AND ENERGY \| METABOLISM AND OBESITY /P] |
| • @MITOCHONDRIAL_LIFE_SCIENCE [MITOCHONDRIAL AND MITOCHONDRIA \| MITOCHONDRIAL AND OXIDATIVE/P] |
| • @MUSCLE_LIFE_SCIENCE [MUSCLE AND SKELETAL \| MUSCLE AND RESISTANCE /P] |
| • @NEUROPEPTIDE_LIFE_SCIENCE [NEUROPEPTIDE AND UNRELATED \| NEUROPEPTIDE AND TREATMENT /P] |
| • @NUCLEUS_LIFE_SCIENCE [NUCLEUS AND ARCUATE \| NUCLEUS AND HYPOTHALAMUS /P] |
| • @OBESE_LIFE_SCIENCE [OBESE AND NORMAL \| OBESE AND OVERWEIGHT /P] |
| • @OBESITY_LIFE_SCIENCE [OBESITY AND INTAKE \| OBESITY AND CONSUMPTION /P] |
| • @OBESOGENIC_LIFE_SCIENCE [OBESOGENIC AND MATERNAL \| OBESOGENIC AND OFFSPRING /P] |
| • @OFFSPRING_LIFE_SCIENCE [OFFSPRING AND MATERNAL \| OFFSPRING AND PREGNANCY /P] |
| • @OIL_LIFE_SCIENCE [OIL AND POINT \| OIL AND OLIVE /P] |
| • @OREXIN_LIFE_SCIENCE [OREXIN AND LATERAL \| OREXIN AND EXCLUSIVELY /P] |
| • @OVERWEIGHT_LIFE_SCIENCE [OVERWEIGHT AND ENERGY \| OVERWEIGHT AND OBESE /P] |
| • @PALATABLE_LIFE_SCIENCE [PALATABLE AND REWARD \| PALATABLE AND OBESITY /P] |
| • @PHYSICAL_LIFE_SCIENCE [PHYSICAL AND ACTIVITY \| PHYSICAL AND VOLUNTARY /P] |
| • @PHYSIOLOGY_LIFE_SCIENCE [PHYSIOLOGY AND COMPARATIVE \| PHYSIOLOGY AND INTEGRATIVE /P] |
| • @PLATYCODIN_LIFE_SCIENCE [PLATYCODIN AND LOWERING \| PLATYCODIN AND CHOLESTEROL /P] |
| • @PREFERENCE_LIFE_SCIENCE [PREFERENCE OR PREFERENCES \| PREFERENCE AND CONDITIONED /P] |
| • @PRODUCTS_LIFE_SCIENCE [PRODUCTS AND RICH \| PRODUCTS AND DAIRY /P] |
| • @PROTEIN_LIFE_SCIENCE [PROTEIN AND MUSCLE \| PROTEIN AND TREATMENT /P] |
| • @RECEPTOR_LIFE_SCIENCE [RECEPTOR OR RECEPTORS \| RECEPTOR AND FAT /P] |
| • @RESISTANCE_LIFE_SCIENCE [RESISTANCE AND INSULIN \| RESISTANCE AND METABOLIC /P] |
| • @RESPONSE_LIFE_SCIENCE [RESPONSE OR RESPONSES & RESPONSE AND TREATMENT /P |
| • @REWARD_LIFE_SCIENCE [REWARD AND PALATABLE \| REWARD AND DOPAMI |
| • @RISK_LIFE_SCIENCE [RISK AND SUGAR \| RISK AND INTAKE /P] |
| • @ROLE_LIFE_SCIENCE [ROLE AND FOOD \| ROLE AND CONSUMPTION /P] |
| • @SATIETY_LIFE_SCIENCE [SATIETY AND INCREASING \| SATIETY AND SATIATIO |
| • @SLEEP_LIFE_SCIENCE [SLEEP AND DECLINES \| SLEEP AND HOURS /P] |
| • @SOFT_LIFE_SCIENCE [SOFT AND ADDICTION \| SOFT AND CARBONATED /P] |
| • @STRESS_LIFE_SCIENCE [STRESS AND OXIDATIVE \| STRESS AND ANTIOXIDAN |
| • @SUCROSE_LIFE_SCIENCE [SUCROSE AND SUGAR \| SUCROSE AND NUCLEUS |
| • @SUGAR_LIFE_SCIENCE [SUGAR AND BEVERAGES \| SUGAR AND RISK /P] |
| • @SWEET_LIFE_SCIENCE [SWEET AND TASTING \| SWEET AND TASTE /P] |
| • @SWEETENED_LIFE_SCIENCE [SWEETENED AND SUGAR \| SWEETENED AND H |
| • @TASTE_LIFE_SCIENCE [TASTE AND SWEET \| TASTE AND SENSORY /P] |
| • @THERMOGENESIS_LIFE_SCIENCE [THERMOGENESIS AND COLD \| THERMOGE |
| • @TISSUE_LIFE_SCIENCE [TISSUE AND ADIPOSE \| TISSUE AND EXPRESSION /P |
| • @VARIETY_LIFE_SCIENCE [VARIETY AND FLAVORS \| VARIETY AND LIMITING /P |
| • @WEIGHT_LIFE_SCIENCE [WEIGHT AND BODY \| WEIGHT AND INTAKE /P] |
| • @ZINC_LIFE_SCIENCE [ZINC AND TRACE \| ZINC AND ELEMENT /P |

**Multidisciplinary Sciences**

| @ACTIVITY_MULTI [ACTIVITY AND PHYSICAL \| ACTIVITY AND LOCOMOTOR /P] |
| --- |
| • @ADVERTISING_MULTI [ADVERTISING AND TELEVISION \| ADVERTISING AND MARKET /P] |
| • @APPETITE_MULTI [APPETITE AND INTAKE \| APPETITE AND PROTEIN /P] |
| • @BEHAVIOR_MULTI [BEHAVIOR OR BEHAVIORS \| BEHAVIOR AND EATING /P] |
| • @BEHAVIOUR_MULTI [BEHAVIOUR OR BEHAVIOURS \| BEHAVIOUR AND PLANNED /P] |
| • @BEVERAGE_MULTI [BEVERAGE AND BEVERAGES \| BEVERAGE AND WATER /P] |
| • @BMI_MULTI [BMI AND BODY \| BMI AND MASS /P] |
| • @BODY_MULTI [BODY AND MASS \| BODY AND INDEX /P] |
| • @BREAKFAST_MULTI [BREAKFAST AND LUNCH \| BREAKFAST AND SKIPPING /P] |
| • @CALORIE_MULTI [CALORIE AND LABELING \| CALORIE AND FINDINGS /P] |
| • @CHANGE_MULTI [CHANGE AND FINDINGS \| CHANGE AND ENVIRONMENT /P] |
| • @CHILDHOOD_MULTI [CHILDHOOD AND *MARKETING* \| CHILDHOOD AND *STAKEHOLDERS* /P] |
| • @CHOICE_MULTI [CHOICE OR CHOICES \| CHOICE AND INCOME /P] |
| • @CONSUMPTION_MULTI [CONSUMPTION AND FOOD \| CONSUMPTION AND INTAKE /P] |
| • @CONTROL_MULTI [CONTROL AND WEIGHT \| CONTROL AND CONSUMPTION /P] |
| • @DAIRY_MULTI [DAIRY AND GRAINS \| DAIRY AND MEAT /P] |
| • @DEVELOPMENT_MULTI [DEVELOPMENT AND METABOLIC \| DEVELOPMENT AND DIET /P] |
| • @DIABETES [DIABETES AND DISEASE \| DIABETES AND COFFEE /P] |
| • @DIETARY_MULTI [DIETARY AND INTAKE \| DIETARY AND CONSUMPTION /P] |
| • @DIET_MULTI [DIET OR DIETS \| DIET AND CONSUMPTION /P] |
| • @ENERGY_MULTI [ENERGY AND INTAKE \| ENERGY AND FOOD /P] |
| • @ENVIRONMENT_MULTI [ENVIRONMENT AND OBESOGENIC \| ENVIRONMENT AND HOME /P] |
| • @ENVIRONMENTAL_MULTI [ENVIRONMENTAL AND EXTERNAL \| ENVIRONMENTAL AND FACTORS /P] |
| • @EXERCISE_MULTI [EXERCISE AND SEDENTARY \| EXERCISE AND STRESS /P] |
| • @FAMILY_MULTI [FAMILY AND EDUCATIONAL \| FAMILY AND MEALS /P] |
| • @FAST_MULTI [FAST AND INFORMATION \| FAST AND MEALS /P] |
| • @FAT_MULTI [FAT AND DIET \| FAT AND CONSUMPTION /P] |
| • @FOOD_MULTI [FOOD OR FOODS \| FOOD AND CONSUMPTION /P] |
| • @FREQUENCY_MULTI [FREQUENCY AND POSITIVE \| FREQUENCY AND POSITIVELY /P] |
| • @FRUIT_MULTI [FRUIT AND VEGETABLES \| FRUIT AND VEGETABLE /P] |
| • @HEALTH_MULTI [HEALTH AND PUBLIC \| HEALTH AND FOODS /P] |
| • @HEALTHY_MULTI [HEALTHY OR UNHEALTHY \| HEALTHY AND EATING /P] |
| • @HOME_MULTI [HOME AND ENVIRONMENT \| HOME AND SOFT /P] |
| • @INCOME_MULTI [INCOME AND HOUSEHOLDS \| INCOME AND PARTICIPATING /P] |
| • @INDEX_MULTI [INDEX AND MASS \| INDEX AND BODY /P] |
| • @INFLUENCE_MULTI [INFLUENCE AND PARENTS \| INFLUENCE AND CONSUMED /P] |
| • @INSULIN_MULTI [INSULIN AND RESISTANCE \| INSULIN AND BLOOD /P] |
| • @INTAKE_MULTI [INTAKE AND FOOD \| INTAKE AND ENERGY /P] |
| • @INTERVENTION_MULTI [INTERVENTION OR INTERVENTIONS \| INTERVENTION AND CARE /P] |
| • @MASS_MULTI [MASS AND INDEX \| MASS AND BODY /P] |
| • @MEAL_MULTI [MEAL OR MEALS \| MEAL AND LUNCH /P] |
| • @METABOLIC_MULTI [METABOLIC AND SYNDROME \| METABOLIC AND FED /P] |
| • @NUTRITION_MULTI [NUTRITION AND KNOWLEDGE \| NUTRITION AND LABELLING /P] |
| • @OBESE_MULTI [OBESE AND OVERWEIGHT \| OBESE AND LEAN /P] |
| • @OBESITY_MULTI [OBESITY AND FOOD \| OBESITY AND CONSUMPTION /P] |
| • @OBESOGENIC_MULTI [OBESOGENIC AND ENVIRONMENT \| OBESOGENIC AND ENVIRONMENTS /P] |
| • @OVERWEIGHT_MULTI [OVERWEIGHT AND OBESE \| OVERWEIGHT AND NORMAL /P] |
| • @PARENTAL_MULTI [PARENTAL AND PARENTS \| PARENTAL AND CHILD /P] |
| • @PARENTS_MULTI [PARENTS AND PARENTAL \| PARENTS AND PRACTICES /P] |
| • @PATTERNS_MULTI [PATTERNS AND DIETARY \| PATTERNS AND ASSOCIATIONS /P] |
| • @PHYSICAL_MULTI [PHYSICAL AND ACTIVITY \| PHYSICAL AND TIME /P] |
| • @POLICY_MULTI [POLICY AND COMMONLY \| POLICY AND MAKERS /P] |
| • @PORTION_MULTI [PORTION AND SIZE \| PORTION AND SIZES /P] |
| • @PRACTICES_MULTI [PRACTICES AND CHILD \| PRACTICES AND FEEDING /P] |
| • @PREVENTION_MULTI [PREVENTION AND EFFECTIVENESS \| PREVENTION AND LIFESTYLE /P] |
| • @PROTEIN_MULTI [PROTEIN AND WHEY \| PROTEIN AND CARBOHYDRATE /P] |
| • @RISK_MULTI [RISK AND FACTORS \| RISK AND BMI /P] |
| • @ROLE_MULTI [ROLE AND PLAY \| ROLE AND EVIDENCE /P] |
| • @SCHOOL_MULTI [SCHOOL OR SCHOOLS \| SCHOOL AND PRIMARY /P] |
| • @SIZE_MULTI [SIZE AND PORTION \| SIZE AND SERVING /P] |
| • @SLEEP_MULTI [SLEEP AND DURATION \| SLEEP AND QUALITY /P] |
| • @SOCIAL_MULTI [SOCIAL AND NORMS \| SOCIAL AND ISOLATION /P] |
| • @SOFT_MULTI [SOFT AND DRINK \| SOFT AND DRINKS /P] |
| • @SUGAR_MULTI [SUGAR AND SWEETENED \| SUGAR AND BEVERAGES /P] |
| • @SWEETENED_MULTI [SWEETENED AND SUGAR \| SWEETENED AND BEVERAGES /P] |
| • @TASTE_MULTI [TASTE AND PREFERENCES \| TASTE AND PERCEPTION /P] |
| • @TELEVISION_MULTI [TELEVISION AND ADVERTISING \| TELEVISION AND VIEWING /P] |
| • @TV_MULTI [TV AND BEDROOM \| TV AND ADVERTISEMENTS /P] |
| • @UNHEALTHY_MULTI [UNHEALTHY OR HEALTHY & UNHEALTHY AND FOODS /P] |
| • @VEGETABLES_MULTI [VEGETABLES AND FRUIT \| VEGETABLES AND FRUITS /P] |
| • @WATER_MULTI [WATER AND BEVERAGE \| WATER AND DOSES /P] |
| • @WEIGHT_MULTI [WEIGHT AND BODY \| WEIGHT AND INTAKE /P |

**Physical Sciences**

| @BADGE_PHYSICAL_SCIENCE [BADGE AND ADIPOGENIC \| BADGE AND ADIPOGENESIS /P] |
| --- |
| • @BMI_PHYSICAL_SCIENCE [BMI AND BODY \| BMI AND MASS /P] |
| • @CHANGE_PHYSICAL_SCIENCE [CHANGE AND CLIMATE \| CHANGE AND PRODUCTION /P] |
| • @CHEMICALS_PHYSICAL_SCIENCE [CHEMICALS AND EXPOSURES \| CHEMICALS AND ENDOCRINE /P] |
| • @COFFEE_PHYSICAL_SCIENCE [COFFEE AND MODERATE \| COFFEE AND MELLITUS /P] |
| • @CONSUMPTION_PHYSICAL_SCIENCE [CONSUMPTION AND FRUIT \| CONSUMPTION AND VEGETABLE /P] |
| • @DENSITY_PHYSICAL_SCIENCE [DENSITY AND RELATION \| DENSITY AND RESIDENTIAL /P] |
| • @DIABETES_PHYSICAL_SCIENCE [DIABETES AND CHEMICALS \| DIABETES AND GUT /P] |
| • @DIET_PHYSICAL_SCIENCE [DIET AND FAT \| DIET AND CONSUMPTION /P] |
| • @ENVIRONMENT_PHYSICAL_SCIENCE [ENVIRONMENT AND FOOD \| ENVIRONMENT AND OBESITY /P] |
| • @EXPOSURE_PHYSICAL_SCIENCE [EXPOSURE AND PRENATAL \| EXPOSURE AND WEIGHT /P] |
| • @FAST_PHYSICAL_SCIENCE [FAST AND OUTLET \| FAST AND OUTLETS /P] |
| • @FAT_PHYSICAL_SCIENCE [FAT AND DIET \| FAT AND EFFECTS /P] |
| • @FOOD_PHYSICAL_SCIENCE [FOOD AND OBESITY \| FOOD AND ENVIRONMENT /P] |
| • @HEALTHY_PHYSICAL_SCIENCE [HEALTHY AND UNHEALTHY \| HEALTHY AND FOODS /P] |
| • @INSULIN_PHYSICAL_SCIENCE [INSULIN AND RESISTANCE \| INSULIN AND METABOLIC /P] |
| • @INTAKE_PHYSICAL_SCIENCE [INTAKE AND FRUIT \| INTAKE AND CONSUMPTION /P] |
| • @LIFE_PHYSICAL_SCIENCE [LIFE AND EARLY \| LIFE AND DISEASE /P] |
| • @NEIGHBOURHOOD_PHYSICAL_SCIENCE [NEIGHBOURHOOD AND WALKABILITY \| NEIGHBOURHOOD AND SUPERMARKETS /P] |
| • @NUTRITION_PHYSICAL_SCIENCE [NUTRITION AND ASSESSMENT \| NUTRITION AND RISK /P] |
| • @OBESOGENIC_PHYSICAL_SCIENCE [OBESOGENIC AND ENVIRONMENTS \| OBESOGENIC AND ENVIRONMENT /P] |
| • @OUTLETS_PHYSICAL_SCIENCE [OUTLETS AND OUTLET \| OUTLETS AND FAST /P] |
| • @OVERWEIGHT_PHYSICAL_SCIENCE [OVERWEIGHT AND CHILD \| OVERWEIGHT AND OBESE /P] |
| • @POPULATION_PHYSICAL_SCIENCE [POPULATION AND HABITS \| POPULATION AND VEGETABLES /P] |
| • @PRODUCTION_PHYSICAL_SCIENCE [PRODUCTION AND CLIMATE \| PRODUCTION AND IMPACTS /P] |
| • @RESISTANCE_PHYSICAL_SCIENCE [RESISTANCE AND INSULIN \| RESISTANCE AND CARBOHYDRATE /P] |
| • @WEIGHT_PHYSICAL_SCIENCE [WEIGHT AND BODY \| WEIGHT AND GAIN /P] |
| • @RISK_PHYSICAL_SCIENCE [RISK AND ASSESSMENT \| RISK AND NUTRITION /P] |

**Social Sciences**

| @ACCESS [ACCESS AND MEASURES \| ACCESS AND OUTLETS /P] |
| --- |
| • @ADVERTISING [ADVERTISING AND REGULATING \| ADVERTISING AND REGULATION /P] |
| • @ATTITUDES [ATTITUDES AND BEHAVIOUR \| ATTITUDES AND ANALYSIS /P] |
| • @AVAILABILITY [AVAILABILITY AND COMPLEX \| AVAILABILITY AND HEALTHY /P] |
| • @BEHAVIOR [BEHAVIOR OR BEHAVIORS \| BEHAVIOR AND ADVERSE /P] |
| • @BEVERAGE [BEVERAGE AND BEVERAGES \| BEVERAGE AND CARBONATED /P] |
| • @BINGE [BINGE AND DISORDER \| BINGE AND PSYCHOSIS /P] |
| • @BMI [BMI AND MASS \| BMI AND BODY /P] |
| • @BODY [BODY AND MASS \| BODY AND INDEX /P] |
| • @CALORIE [CALORIE OR CALORIES \| CALORIE AND INTERACTIONS /P] |
| • @CITY [CITY AND DEVELOPMENT \| CITY AND COMBAT /P] |
| • @CONSUMPTION [CONSUMPTION AND FOOD \| CONSUMPTION AND FOODS /P] |
| • @CONTRACTS [CONTRACTS AND ADVERTISEMENTS \| CONTRACTS AND AGRICULTURE /P] |
| • @CONTROL [CONTROL AND PROPOSED \| CONTROL AND GEOGRAPHICALLY /P] |
| • @CORTISOL [CORTISOL AND STRESS \| CORTISOL AND PROBLEMS /P] |
| • @CUES [CUES OR CUE & CUES AND SIGNALING /P] |
| • @DIET [DIET AND HIGHER \| DIET AND FREQUENCY /P] |
| • @DIETARY [DIETARY AND DIET \| DIETARY AND FAT /P] |
| • @DIETERS [DIETERS AND DIETING \| DIETERS AND FACILITATE /P] |
| • @DRINK [DRINK OR DRINKS \| DRINK AND SOFT /P] |
| • @EARLY_SOCIAL_SCIENCE [EARLY AND FEEDING \| EARLY AND LIFE /P] |
| • @EMOTIONAL [EMOTIONAL OR EMOTIONS \| EMOTIONAL AND NEGATIVE /P] |
| • @EMPLOYMENT [EMPLOYMENT AND POLITICAL \| EMPLOYMENT AND DEVELOPMENT /P] |
| • @ENERGY [ENERGY AND INTAKE \| ENERGY AND EXPOSURE /P] |
| • @FAST [FAST AND OUTLETS \| FAST AND RESTAURANTS /P] |
| • @FAT [FAT AND MEASURED \| FAT AND ENERGY /P] |
| • @FOOD [FOOD OR FOODS \| FOOD AND CONSUMPTION /P] |
| • @HEALTH [HEALTH AND FOOD \| HEALTH AND OBESITY /P] |
| • @HEALTHY_SOCIAL_SCIENCE [HEALTHY AND EATING \| HEALTHY AND VEGETABLES /P] |
| • @HOME [HOME AND PARTICIPATING \| HOME AND BELIEFS /P] |
| • @INCOME [INCOME AND STATUS \| INCOME AND HOUSEHOLDS /P] |
| • @INTAKE [INTAKE AND ENERGY \| INTAKE AND EXPENDITURE /P] |
| • @INTERVENTION [INTERVENTION AND CARBOHYDRATES \| INTERVENTION AND CHOICES /P] |
| • @LIFESTYLE [LIFESTYLE AND CONSUMER \| LIFESTYLE AND VARIABLES /P] |
| • @MENU [MENU AND RESTAURANT \| MENU AND REGULATORY /P] |
| • @MOOD [MOOD AND CONDITION \| MOOD AND NEGATIVE /P] |
| • @NUTRITION [NUTRITION AND DIET \| NUTRITION AND PATTERNS /P] |
| • @OBESITY [OBESITY AND CHILDHOOD \| OBESITY AND CONSUMPTION /P] |
| • @OVERWEIGHT [OVERWEIGHT AND WEIGHT \| OVERWEIGHT AND OBESE /P] |
| • @PACKAGES [PACKAGES AND SMALL \| PACKAGES AND LARGE /P] |
| • @PALATABLE [PALATABLE AND RECEPTORS \| PALATABLE AND CHRONIC /P] |
| • @POLICY [POLICY AND CHALLENGE \| POLICY AND POLICIES /P] |
| • @POPULATION [POPULATION AND LEVEL \| POPULATION AND LOCAL /P] |
| • @PRICE [PRICE OR PRICES & PRICE AND EFFECTS /P] |
| • @SATIATION [SATIATION AND SATIATE \| SATIATION AND TYPICALLY /P] |
| • @SCHOOL [SCHOOL AND NUTRITIONAL \| SCHOOL AND CHILDREN /P] |
| • @SIZE [SIZE AND LARGER \| SIZE AND SMALLER /P] |
| • @SMALL [SMALL AND PACKAGES \| SMALL AND LARGE /P] |
| • @SNACK [SNACK OR SNACKS \| SNACK AND UNHEALTHY /P] |
| • @SOFT [SOFT AND DRINKS \| SOFT AND DRINK /P] |
| • @STRESS [STRESS AND URBANISATION \| STRESS AND TYPICALLY /P] |
| • @SUCROSE [SUCROSE AND FAT \| SUCROSE AND DOPAMINE /P] |
| • @SUPERMARKET [SUPERMARKET AND SIZES \| SUPERMARKET AND AFFORDABILITY /P] |
| • @TAX [TAX OR TAXES \| TAX AND FOOD /P] |
| • @WEIGHT [WEIGHT AND OVERWEIGHT \| WEIGHT AND DIFFERENCES /P |
